# O03 - Expression of pulmonary surfactant protein D (SP-D) and interleukin 13 in the serum of atopic and non-atopic severe pediatric asthmatics: effects of glucocorticoid and sodium cromoglycate treatment

**DOI:** 10.1186/2045-7022-4-S1-O3

**Published:** 2014-03-14

**Authors:** Vasiliki Gemou-Engesaeth, Nikoletta Laliotou, Chris J Corrigan, George Chrousos, Angela Haczku

**Affiliations:** 1 National and Kapodistrian University of Athens, Athens, Greece; 2 Guy’s, King’s and St. Thomas’ School of Medicine, London, United Kingdom; 3 University of Pennsylvania, Philadelphia, PA, USA

## Rationale

Serum biomarker analysis is a valuable noninvasive tool for assessment of asthma severity in children. Surfactant protein D (SP-D) is an immunoprotective lung collectin produced in the lung in large quantities. We have previously shown that allergen-induced airway inflammation was associated with significant changes of SP-D release and that glucocorticoid therapy increases SP-D in the lung. Whether such changes could be detected in the peripheral circulation of asthmatic patients is not known. We hypothesized that serum SP-D would reflect disease severity in asthmatic children.

## Methods

SP-D and IL-13 serum levels were measured in 122 patient samples and 22 samples from non-asthmatic controls. Serum was collected from peripheral blood of 25 children with asthma (diagnosed according to the American Thoracic Society criteria), that was inadequately controlled according to European Consensus Guidelines, as described previously by Gemou-Engesaeth et al., 2002. Children were characterized as atopic (17) and as nonatopic (8). Their age was between 7 and 16 years. In addition 15 nonasthmatic controls matched for age and atopic status were included in the study. SP-D was assessed in duplicate samples in two dilution (1:5 and 1:10) using a commercially available human SP-D ELISA kit (BioVendor). IL-13 was assessed using a kit from RayBiotech, Inc.Measurements were repeated in 17 of the asthmatics 4 to 6 months after initiation or escalation of inhaled glucocorticoid therapy (in 10) and after initiation of inhaled Sodium Cromoglycate(in 7) for inadequately controlled asthma.

## Results

The inter-assay and inter-experimental variability of the measures was <10%. IL-13 levels varied between 0-171 pg/ml. SP-D levels varied between 19-373 ng/ml. The expression of IL-13 was significantly greater in the asthmatic serum samples than in the controls (25.6±2.7 vs. 14.3±2.0 p=0.0011, 2-tail t test, unequal variability). In contrast, SP-D levels were significantly greater in the control samples than in the asthmatic samples (148.2±5.9 vs. 200.2±20.2; p=0.0478). Treatment with glucocorticoids or sodium cromoglycate did not affect IL-13 or SP-D in the serum although in the sodium cromoglycate treated patients there was a trend for reduced IL-13.

## Conclusions

These data indicate that serum levels of IL-13 and SP-D correlate with the presence of allergic airways disease in children. We speculate that these biomarkers may provide useful noninvasive indicators that reflect disease state in moderate to severe childhood asthma.

## Funding

GSK, Rhône-Poulenc Rorer, The Norwegian Asthma and Allergy Association, NAHR, R01AI072197(AH); RC1ES018505(AH); P30ES013508(AH).

